# Electrical stimulation for limb spasticity in children with stroke: A protocol for systematic review

**DOI:** 10.1097/MD.0000000000021042

**Published:** 2020-07-02

**Authors:** Jing Nie, He Wang, Quan-wei Jiang, Ying Zhang, Zhi-guang Zhang, Mei Mei

**Affiliations:** a First Ward of Pediatrics Department; b Department of Neurosurgery, First Affiliated Hospital of Jiamusi University, Jiamusi; c Department of Anesthesiology, Benxi Central Hospital, Benxi; d Department of Neurology, Third Affiliated Hospital of Heilongjiang University of Traditional Chinese Medicine, Harbin; e Department of Neurology, Hongda Hospital of Jiamusi University, Jiamusi, China.

**Keywords:** effectiveness, electrical stimulation, limb spasticity, safety, stroke

## Abstract

**Background:**

This systematic review protocol will appraise the effectiveness and safety of electrical stimulation (ES) for limb spasticity (LS) in children with stroke.

**Methods:**

Cochrane Library, EMBASE, PUBMED, PsycINFO, Scopus, OpenGrey, CINAHL, ACMD, CNKI, and WANGFANG will be systematically retrieved for randomized controlled trials (RCTs) testing the effectiveness of ES compared with other interventions on LS in children with stroke. Two independent authors will evaluate eligibility using predefined criteria and will perform data extraction and study quality appraisal of eligible trials. Primary outcomes include gait velocity, and limb spasticity status. Limb function, quality of life, pain intensity, and adverse events will be assessed as secondary outcomes. We will perform data analysis using RevMan 5.3 software.

**Results:**

This systematic review will summarize the most recent evidence to assess the effectiveness and safety of ES for LS in children with stroke.

**Conclusions:**

The results of this study may help to determine whether ES is effective or not for LS in children with stroke.

**Study registration:**

INPLASY202050115.

## 1 Introduction

Stroke is a leading cause of death and disability around the world.^[1–3]^ Most stroke survivors often develop motor dysfunction and limb spasticity,^[4,5]^ which significantly limit their mobility and functional ability, and thus affect quality of life.^[6–8]^ It has been estimated that its incidence varies from 17% to 38%.^[9–11]^ Given such severe conditions, effective treatments are necessary to manage their rehabilitation. However, few effective interventions have been developed in treating spasticity post stroke, especially in children population.^[12–16]^

Previous studies have reported that electrical stimulation (ES) has been found to decrease limb spasticity (LS) following stroke.^[17–27]^ However, no systematic review supports the use of ES for LS in children after stroke. Thus, the present systematic review protocol will target to evaluate the effectiveness and safety of ES for relieving post stroke LS in children population.

## 2 Methods and analysis

### 2.1 Study registration

This study was registered at INPLASY (INPLASY202050115), and it is reported following the Preferred Reporting Items for Systematic Reviews and Meta-Analysis Protocol statement guidelines.^[28]^

### 2.2 Ethics and dissemination

This study will not require ethic approval, because it will be conducted based on published studies. We will publish this study on a peer-reviewed journal or relevant conference.

## 3 Inclusion criteria for study selection

### 3.1 Type of studies

This systematic review will consider randomized controlled trials (RCTs) on effectiveness and safety of ES for LS in children with stroke for inclusion. We will exclude animal study, review, editorial letter, comment, case report, case series, uncontrolled trial, and quasi-RCTs.

### 3.2 Type of participants

All children under 18 years old with LS following stroke will be included, in spite of ethnicity, country, and severity of LS and stroke.

### 3.3 Type of interventions

In the experimental group, all patients received any types of ES, such as neuromuscular electrical stimulation, transcutaneous electrical nerve stimulation, and electroacupuncture.

In the control group, no restrictions will be applied to any comparators. However, we will not consider any types of ES.

### 3.4 Type of outcomes

Primary outcomes are gait velocity (as assessed by Gait Velocity Assessment Toolkit or other scales), and limb spasticity status (as evaluated by Modified Ashworth Scale or other tools).

Secondary outcomes are limb function (as appraised by Disability Assessment Scale or other scales), quality of life (as detected by 36-Item Short Form Survey or other surveys), pain intensity (as measured by Visual Analogue Scale or other scales), and adverse events.

### 3.5 Search strategy

A systematic search will be performed from inception to the present without language and publication status limitations in Cochrane Library, EMBASE, PUBMED, PsycINFO, Scopus, OpenGrey, CINAHL, ACMD, CNKI, and WANGFANG. All eligible RCTs testing the effectiveness and safety of ES on LS in children with stroke will be included. We will build detailed search strategy for Cochrane Library in Table 1, and will adapt similar retrieval strategies for other electronic databases. In addition, this study will examine other sources, such as conference information, ongoing, or unpublished studies from clinical trial registry, and reference lists of relevant reviews.

**Table 1:**
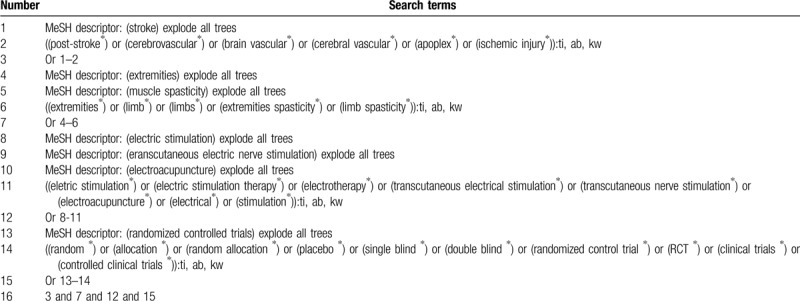
Search strategy for Cochrane Library.

## 4 Data collection and management

### 4.1 Study selection

Two independent authors will import all searched citations into EndNote X8 to remove duplicates. At first, we will read titles/abstracts to eliminate any irrelevant record. Then, we will carefully identify full-text of remaining potential studies against all eligibility criteria. Any doubt will be clarified by a third author through discussion and consensus. Studies excluded will be noted with reasons for their exclusion. We will present selection of study procedure in a flow diagram.

### 4.2 Data extraction and management

Using a predefined and developed data extraction form, 2 independent authors will extract data from all eligible RCTs. Any disagreement will be solved by a third author via discussion. We will extract following information: first author, journal/source, country, year of publication, trial design, setting, sample size, diagnostic criteria, inclusion/exclusion criteria, details of ES and controls, outcomes, adverse events, results, findings, follow-up information, and conflict of interest.

### 4.3 Missing data dealing with

If we identify any insufficient or unclear or missing data, we will obtain them by contacting primary authors through email. We will analyze available data if we can not obtain that data.

### 4.4 Study quality assessment

Two independent authors will appraise study quality for eligible RCTs using Cochrane Risk of Bias Tool with predetermined criteria. Each study will be rated as a high, unclear or low risk of bias. Any divergence will be solved by a third author through consensus.

### 4.5 Statistical analysis

We will utilize RevMan 5.3 software to pool and analyze data. All dichotomous outcomes will be estimated as relative risk/risk ratio with 95% confidence intervals (CIs); and continuous outcomes will be calculated as weighted mean difference with 95% CIs. Statistical heterogeneity will be examined using *I*^*2*^ test. Values of *I*^*2*^ are less than 50% will be considered as minor heterogeneity, while *I*^*2*^ values over 50% will be suggested as significant heterogeneity. We will carry out meta-analysis if minor heterogeneity is tested and sufficient data is extracted. Otherwise, we will perform descriptive analyses for those studies which are deemed clinically heterogeneous or aggregate data for synthesizing.

### 4.6 Subgroup analysis

Where applicable, we will conduct subgroup analysis or meta-regression for factors presumed to cause significant heterogeneity or variations in study characteristics, details of interventions and controls, and outcome indicators.

### 4.7 Sensitivity analysis

Whenever possible, we will perform sensitivity analysis to test robustness and stability of study findings based on study quality, sample size and missing, or insufficient data.

### 4.8 Reporting bias

When over 10 RCTs are included, we will check reporting bias using funnel plot,^[29]^ and Egger linear regression test.^[30]^

## 5 Discussion

LS is one of the most common complications in post stroke survivors. Although ES is utilized in the clinical treatment of LS, its effectiveness and safety in children with stroke continues to be debated. Recent studies have provided increasing evidence in this field; however, no systematic review has addressed this issue. The present study will summarize high quality RCTs to evaluate the effectiveness and safety of ES in treating LS in children following stroke. The results of this study will help to determine whether ES is effective or not for the treatment of LS in children with stroke, which may benefit both patients and clinical practice.

## Author contributions

**Conceptualization:** Jing Nie, He Wang, Quan-wei Jiang.

**Data curation:** He Wang, Ying Zhang, Zhi-guang Zhang.

**Formal analysis:** Jing Nie, Quan-wei Jiang, Ying Zhang.

**Methodology:** Jing Nie, Quan-wei Jiang, Ying Zhang, Zhi-guang Zhang.

**Resources:** Jing Nie, He Wang, Quan-wei Jiang, Ying Zhang, Zhi-guang Zhang.

**Software:** Jing Nie, He Wang, Quan-wei Jiang, Ying Zhang, Zhi-guang Zhang.

**Validation:** Jing Nie, He Wang, Ying Zhang, Zhi-guang Zhang.

**Visualization:** Jing Nie, Quan-wei Jiang, Ying Zhang.

**Writing – original draft:** Jing Nie, He Wang, Ying Zhang, Zhi-guang Zhang.

**Writing – review & editing:** Jing Nie, Quan-wei Jiang.
